# National Malaria Control Program in Bangladesh: 2007-2010

**DOI:** 10.7759/cureus.35899

**Published:** 2023-03-08

**Authors:** Afsana A Khan, Mohammad J Karim

**Affiliations:** 1 Communicable Disease Control Department, Directorate General of Health Services, Dhaka, BGD; 2 Public Health Department, Nilphamari 250 Bed General Hospital, Directorate General of Health Services, Nilphamari, BGD

**Keywords:** bangladesh, incidence, morbidity, mortality, malaria

## Abstract

Malaria is a life-threatening disease caused by the plasmodium parasite, which is transmitted to people by the bites of infected mosquitoes. As elsewhere, this disease is also a major public health problem in Bangladesh. After independence, dichlorodiphenyltrichloroethane (DDT) use was banned in 1985, and the number of malaria cases began to increase. There were no control programs and inadequate funds, especially in the malaria-endemic areas; thus, malaria cases started to be epidemic in the 1990s. The global fund has been supporting the National Malaria Control Program (NMCP) in Bangladesh since the approval of the round 6 malaria proposal in 2006. This study aims to review the NMCP and changes in the burden of malaria in Bangladesh from 2007 to 2010. This is a descriptive retrospective study based on the secondary malaria surveillance data (cases and deaths) in 13 malaria-endemic districts, especially five selected districts, Chittagong, Cox’s Bazar, Rangamati, Sylhet, and Mymensingh. A descriptive analysis was carried out to establish the incidence and mortality rate. From 2007 to 2010, a total of 264,293 confirmed malaria cases were notified from 13 malaria-endemic districts. More than 50% of the affected population was under the age group of ≥15 years (55.7%). Males had a higher risk of contracting of malaria than females, accounting for 53.5% of confirmed cases compared to 46.5% of females. Among the affected population, *Plasmodium falciparum* caused 85.6% of the total incidence. Rangamati has the highest incidence rate among the five districts. Although the incidence was high, death was declining: in 2007, it was 228, and in 2010, it was 37. The finding shows that while the incidence is still high, mortality is decreasing, therefore, it can be said that the NMCP is functioning. However, to fully achieve the goal of eliminating malaria, the NMCP requires efforts to develop new strategies and maintain a high-quality surveillance and reporting system.

## Introduction

Malaria is one word, but its impact is throughout the world. It has a worldwide history of 400 years. It is still one of the biggest threats to world health in terms of morbidity, mortality, and the developing economy. This life-threatening disease is caused by five species of parasites of the genus Plasmodium that affect humans (*Plasmodium falciparum*, *P. ovale*, *P. malariae*, *P.​​​​​​​ vivax, *and *P.​​​​​​​ knowlesi*) [1]. Malaria due to *P. falciparum *is the most deadly and predominates in Africa. Although residents of Sub-Saharan Africa have the highest risk of developing malaria of all geographical locations, an estimated 3.3 billion people were at risk in 2010; in 2010, 81% of cases and 91% of deaths were estimated to have occurred in the World Health Organization (WHO) African Region, with children under five years of age and pregnant women being most severely affected [1]. In 2000, malaria was estimated to contribute to the loss of nearly 45 million disability-adjusted life years (DALYs), which represents about 13% of all infectious diseases [2]. Malaria is an entirely preventable and treatable disease, provided that currently recommended interventions are properly implemented. It included (i) vector control using insecticide-treated nets (ITNs), indoor residual spraying (IRS), and in some circumstances, larval control; (ii) chemoprevention for the most vulnerable groups, especially pregnant women and newborns; (iii) rapid diagnostic tests (RDTs) or microscopy to determine the diagnosis of malaria in each suspected case; and (iv) prompt administration of proper anti-malarial medications (according to the parasite species and any documented drug resistance) [1].

Bangladesh has a serious public health issue with malaria. Ten of the 11 nations in the South-East Asian Regional Office of the World Health Organization, including Bangladesh, have an endemic malaria problem. Prior to 1971, malaria was largely under control as a result of the frequent use of dichlorodiphenyltrichloroethane (DDT) by the East Pakistani government's program to eradicate the disease [3]. DDT was outlawed in 1985, following Bangladesh's separation from Pakistan, and a rise in malaria incidence followed. In Bangladesh's malaria-endemic areas, there were no ongoing control measures because the incidence of malaria in the eastern regions was low, and there were insufficient resources and programs. In the 1990s, malaria cases began to rise and spread like an epidemic without these management measures [3,4]. In the late 1990s, more than 500 malaria fatalities, 70,000 laboratory-confirmed cases, and 900,000 clinical cases of malaria were recorded in Bangladesh [5].

The majority of these occurrences take place in the 13 districts that are near or border Myanmar and/or India. Out of Bangladesh's 64 districts, 13 have been identified as having an endemic malaria problem. Ninety-eight percent of the malaria case reports come from these 13 districts. The Global Fund to Fight Aids, Tuberculosis and Malaria (GFATM) has been supporting the National Malaria Control Program (NMCP) in Bangladesh since the approval of the round 6 malaria proposal in 2006. The Global Fund-supported malaria program in Bangladesh is currently being implemented under a dual-track funding mechanism through a consortium of a wide network of over 20 private and non-governmental organizations in these 13 malaria-endemic districts of Bangladesh. The target of the NMCP is to decrease the overall malaria burden (morbidity and mortality) among the 10.9 million people who live in these 13 hyperendemic districts of Bangladesh by 60% by 2015 compared to the previous year of 2008 [6].

According to the 2011 World Malaria Report, 106 countries are within this NMCP to achieve a “malaria-free world.” Their targets are now, by the end of 2015, to (i) reduce global malaria deaths to near zero, (ii) reduce global malaria cases by 75% from 2000 levels, and (iii) eliminate malaria in 10 new countries since 2008, including in the WHO European Region [7]. After this program began, mortality and morbidity started to decline in most of the countries, and in October 2011, Armenia was certified free of malaria by the World Health Organization (WHO) [8]. This study aimed to present the changes that have occurred in malaria prevalence, morbidity and mortality from 2007 to 2010, following the implementation of the NMCP in Bangladesh.

## Materials and methods

This is a retrospective descriptive study. The data were secondary recorded data from 2007 to 2010. They were collected from the 13 malaria-endemic areas (districts). The majority of the districts were located around the eastern and northern borders of India and Myanmar. These areas were mostly forest and hilly areas. Five districts were chosen from the 13 districts: four from hilly areas and one from plains. These are Chittagong, Rangamati, Cox’s Bazar, Sylhet, and Mymensingh. The malaria data includes the annual number of people who were tested in the local hospital. Fever or a history of fever during the previous 48 hours, no other symptoms of sickness, and insufficient anti-malarial treatment (or none at all) over the four weeks before to the current illness were the diagnostic criteria. The district health system is used to carry out malaria control initiatives at the district level and beyond. Each upazila within a district contains six to ten unions, and each union is further subdivided into nine wards (blocks). A 31-50 bed hospital (the most peripheral level of inpatient institution) and a group of field workers up to the ward level are located in each upazila. One health administrator, nine doctors, a senior staff nurse, laboratory technicians, supervisory personnel, and field employees make up the upazila health team. To provide curative, preventative, and promotional healthcare services to an average of 250,000 people within its catchment area, the upazila health team is crucial. There are union health clinics (static outpatient facilities) in each upazila to serve the 25,000-30,000 residents there. Some of the union-level static health facilities are administered by paramedical staff, while others are staffed with medical professionals (medical assistants). Microscopy-based diagnosis is accessible up to the sub-district level but is not always available. Rapid diagnostic test (RDT) for falciparum has also been made available in the nation. The RDT is used in the community to diagnose malaria cases by both government and non-governmental organization (NGO) personnel. For the purpose of implementing the operations of the disease control program at the community level, the upazila health complexes (UHCs) have a cadre of field employees in addition to their direct supervisors. All field implementations, including the malaria control program, are the UHCs' responsibility. The accountable management in the upazila is the health and family planning officer. Similarly to this, the district authority oversees all district-level program operations and reports to the Directorate General of Health Services (DGHS).

Bangladesh secured a Round-6 Global Fund to Fight Aids, Tuberculosis, and Malaria (R-6 GFATM) grant and is currently putting into practice a concept that focuses on encouraging the use of insecticide-treated nets and long-lasting insecticide-treated nets (ITNs and LLINs) and early diagnosis of uncomplicated *P. falciparum *malaria by introducing RDT with enhanced microscopy. Day of diagnosis, age, gender, ward number, upazila health complex name, malaria spice type, incidence, and mortality were the factors that made up the data.

## Results

Malaria case notification, incidence rate and mortality from 2007 to 2010, a total of 264,293 confirmed malaria cases were notified in 13 malaria-endemic districts of Bangladesh. Table 1 demonstrated malaria incidence according to age, gender, and Plasmodium species. All patients affected were aged 1 to >15 years age group and all age groups were affected, 5708 (2.2%) from below one year, 34,849 (13.2%) from 1 to 4-year group, and 76,649 (29.0%) from 5 to 14 years age group. Our study findings show that more than 50% of the affected population is in the age group of ≥15 years (55.7%). Males had a higher risk of contracting malaria than females, accounting for 53.5% of confirmed cases compared to 46.5% of females. Malaria incidence according to *P. falciparum* (PF) and *P. vivax* (PV) is shown in Table 1. The overall incidence showed that among the affected population, 226,141 (85.6%) were affected by *P. falciparum* (PF), 38,152 (14.4%) were affected by *P. vivax *(PV) (Table 1). 

**Table 1:** Malaria incidence (age, gender, and Plasmodium species). PF: *P. falciparum*, PV: *P. vivax.*

	2007 n (%)		2008 n (%)		2009 n (%)		2010 n (%)		All n (%)	
Age group										
<1	2237	(3.74)	2213	(2.61)	737	(1.15)	521	(0.93)	5708	(2.2)
1-4	10,459	(17.47)	11,481	(13.56)	6633	(10.38)	6276	(11.23)	34,849	(13.2)
5-14	19,695	(32.90)	24,878	(29.38)	17,019	(26.65)	15,057	(26.95)	76,649	(29.0)
≥15	27,466	(45.89)	46,118	(54.46)	39,484	(61.82)	34,019	(60.89)	147,087	(55.7)
Total	59,857	(100)	84,690	(100)	63,873	(100)	55,873	(100)	264,293	(100)
Gender										
Male	29,865	(49.89)	44,984	(53.12)	34,988	(54.78)	31,669	(56.68)	141,506	(53.5)
Female	29,992	(50.11)	39,706	(46.88)	28,885	(45.22)	24,204	(43.32)	122,787	(46.5)
Total	59,857	(100)	84,690	(100)	63,873	(100)	55,873	(100)	264,293	(100)
Plasmodium species										
PF	46,791	(78.17)	70,281	(82.99)	57,020	(89.27)	52,049	(93.16)	226,141	(85.6)
PV	13,066	(21.83)	14,409	(17.01)	6853	(10.73)	3824	(6.84)	38,152	(14.4)
Total	59,857	(100)	84,690	(100)	63,873	(100)	55,873	(100)	264,293	(100)

**Figure 1:**
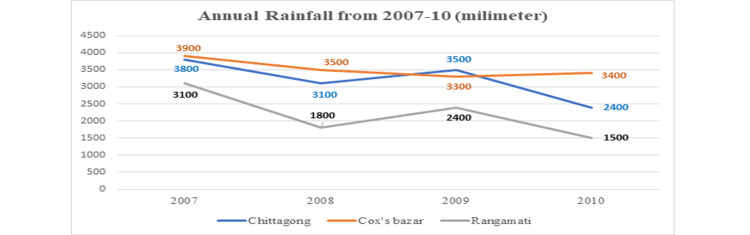
Annual rainfall from 2007 to 2010.

Figure 1 shows the annual rainfall from 2007 to 2010. In 2007, 3800% were in Chittagong, 3900% were in Cox’s Bazar, and 3100% were in Rangamati. In 2008, 3100% were in Chittagong, 3500% were in Cox’s Bazar, and 1800% were in Rangamati. In 2009, 3500% were in Chittagong, 3300% were in Cox’s Bazar, and 2400% were in Rangamati. In 2010, 2400% were in Chittagong, 3400% were in Cox’s Bazar, and 1500% were in Rangamati.

**Figure 2:**
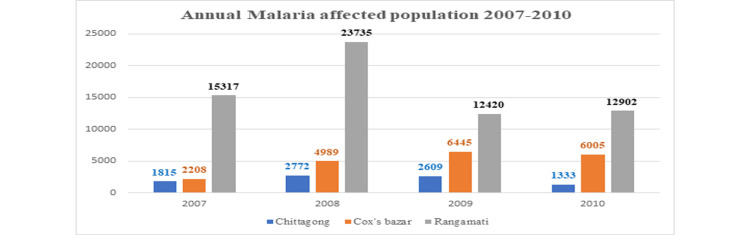
Annual malaria-affected population 2007-2010.

Figure 2 shows the annual malaria-affected population for 2007-2010. In 2007, 1815 were affected in Chittagong, 2208 were affected in Cox’s Bazar, and 15,317 were affected in Rangamati. In 2008, the notably increased in the Rangamati district, with 23,735 people affected, and then gradually decreased in the next year. In 2010, there was decreased number in these three regions. 

**Figure 3:**
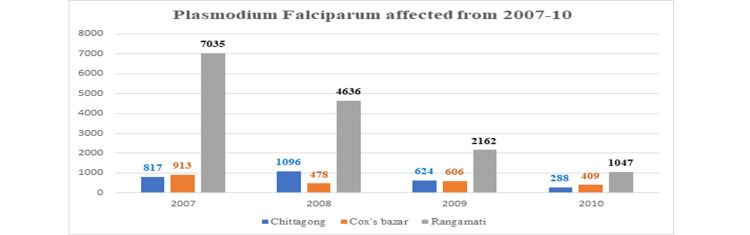
Plasmodium falciparum affected from 2007 to 2010 in Chittagong, Cox’s Bazar, and Rangamati districts.

This study found a high incidence of *P. falciparum *in Rangamati in all years (Figure 3).

**Figure 4:**
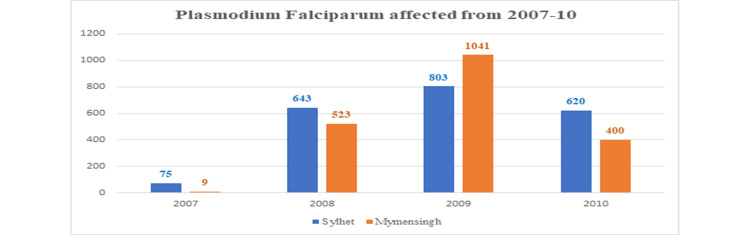
Plasmodium falciparum affected from 2007 to 2010 in Sylhet and Mymensingh districts.

In 2007, there was a low incidence, with 75 cases affected in Sylhet and only nine cases affected in Mymensingh by *P. falciparum*. Then increased in the next two years and again decreased in 2010 (Figure 4).

**Figure 5:**
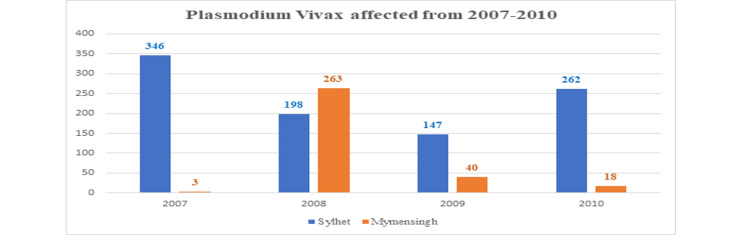
Plasmodium vivax affected from 2007 to 2010.

This result is indicating that the National Malaria Control Program (NMCP) is functioning (Figure 5). 

**Figure 6:**
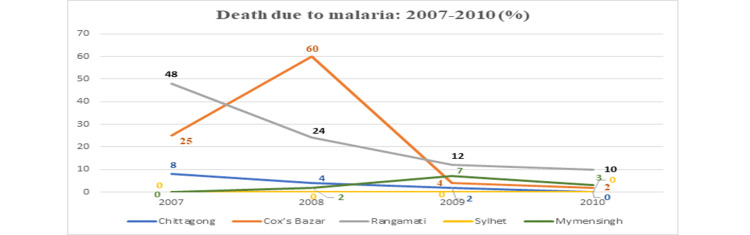
Death due to malaria from 2007 to 2010.

We found a decreased mortality rate. Initially, Rangamati (48%) had a high mortality rate in 2007, and then reports showed a lower rate gradually. There was a peak (60%) in Cox’s Bazaar in 2008. Low mortality rates are found in Chittagong and Mymensingh districts. No death reports were found in Sylhet (Figure 6).

Here according to monthly data of temperature minimum, temperature maximum, rainfall, and humidity of mean were 13.625°C, 26.460°C, 9.40 mm, 75.80%, and ±SD were 1.526°C, 2.142°C, 17.605 mm, and 5.177% (Table 2).

**Table 2:** Mean and standard deviation for average/monthly data from 2007 to 2010.

Monthly data	Mean	±SD
Temperature minimum	13.625°C	1.526°C
Temperature maximum	26.460°C	2.142°C
Rainfall	9.40 mm	17.605 mm
Humidity	75.80%	5.177%

There was a higher incidence in Cox’s Bazaar, Rangamati, and Chittagong districts and a lower incidence in Mymensingh and Sylhet. The total incidence in 2007, 2008, 2009, and 2010 was, accordingly, 28,538, 29,274, 32,620, and 33,403 (Table 3).

**Table 3:** Distribution of the study according to the incidence of different districts.

Year	Population	Total incidence	Incidence in district absolute and %				
			Chittagong, n (%)	Cox’s Bazar, n (%)	Sylhet, n (%)	Mymensingh, n (%)	Rangamati, n (%)
2007	9,343,266	28,538	2632 (0.05)	3121 (0.17)	421 (0.00)	12 (0.00)	22,352 (3.99)
2008	9,343,266	29,274	3368 (0.06)	6467 (0.36)	841 (0.10)	795 (0.14)	28,371 (5.06)
2009	9,343,266	32,620	3233 (0.06)	7051 (0.39)	950 (0.11)	1081 (0.18)	14,582 (2.60)
2010	9,343,266	33,403	1621 (0.03)	6414 (0.36)	882 (0.11)	418 (0.07)	13,949 (2.49)

**Figure 7:**
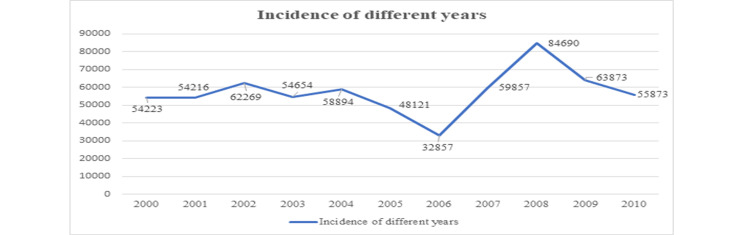
Line graph of the study according to the incidence of different years.

The incidence of different years (2000-2010) has also been shown in Figure 7.

**Figure 8:**
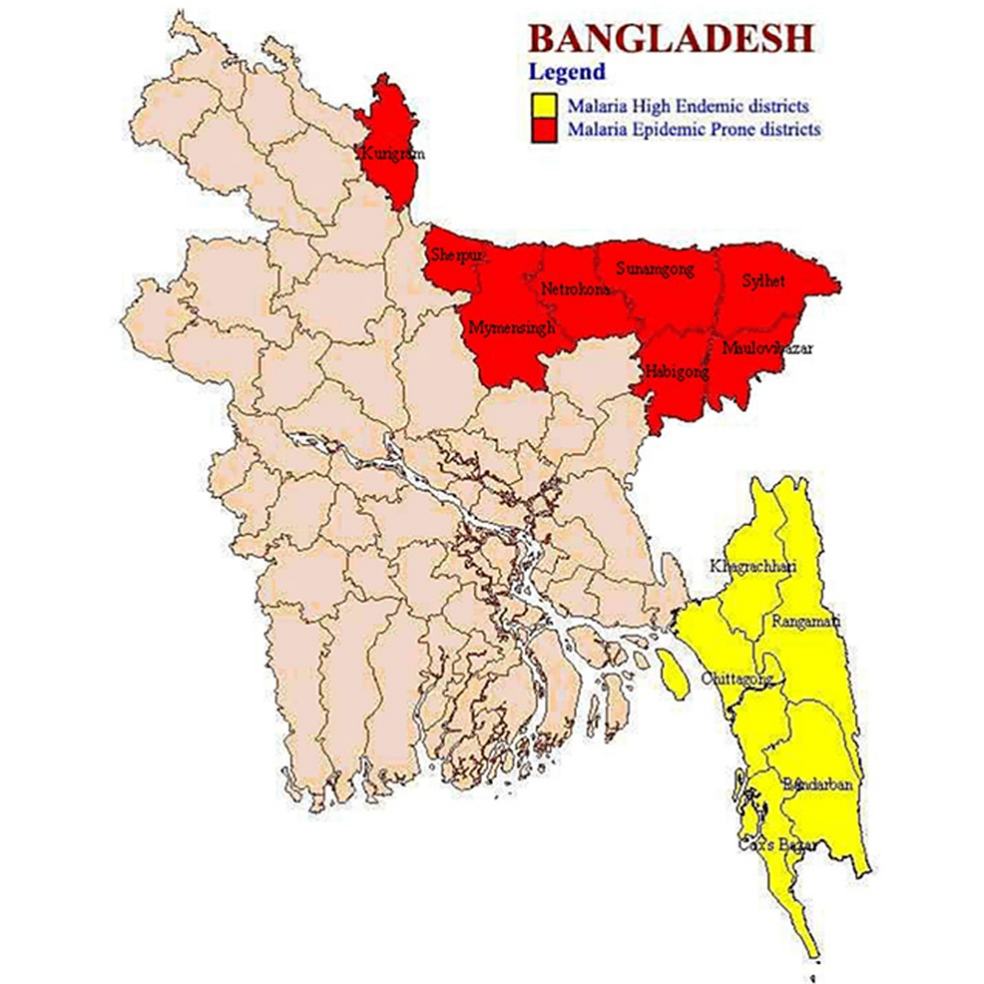
Malaria high-endemic districts and malaria epidemic-prone districts in Bangladesh.

Malaria high-endemic districts and malaria epidemic-prone districts in Bangladesh had been shown in a map (Figure 8). In Bangladesh, malaria high-endemic districts were Kurigram, Sherpur, Mymensingh, Netrokona, Sunamgong, Habigong, Maulovibazar, and Sylhet. Malaria epidemic-prone districts were Khagrachuri, Rangamati, Chittagong, Bandarban, and Cox’s Bazar.

## Discussion

The trend showed that malaria mortality and morbidity gradually decreased in Bangladesh from 2007 to 2010. A previous study in Mpumalanga province in South Africa also showed the same sort of trend [9]. In 2007, malaria caused deaths were 228, and in 2010, the number decreased to 37 in Bangladesh. A notable rise in the number of notified causes was observed in 2008, and it might be a consequence of NMCP's new policies aimed at preventing parasite resistance [10]. Like providing LLINs, improving diagnostic methods like RDT, increasing awareness among people about malaria and how it is transmitted and its pathogenesis by providing health education. Peak malaria incidence (55.7%) in the young adult age group (15 years) in the current study may be associated to outdoor behavioral risk variables including leisure and sleeping habits as well as outdoor work leading to exposure to infective mosquito bites [11-14].

This study also found that malaria incidence was high among males (53.5%). This may be attributed to the fact that men are significantly more likely to get malaria due to their profession, particularly those who work in the fields, forests, or travel to regions with high malaria endemicity [15]. Evidence of the connection between work and malaria risk comes from a study done in Ethiopia. They discovered that migratory workers were more susceptible to malaria in highland endemic regions [16].

In this study, it was found that *P. falciparum* (85.6% of total incidence) has very high transmission in the highlands like Chittagong, Cox’s Bazar, and Rangamati when compared with Sylhet and Mymensingh. But overall, the situation shows us that *P. falciparum* is the main species that causes malaria in Bangladesh. Other studies also show that an estimated 271 million *P. falciparum* cases in 47 countries on the African continent and 180 million *P. falciparum* cases in other countries during 2007 occurred [17]. One study in India also showed that most of the malaria cases reported were attributed to *P. falciparum* (88.3%) [18]. In Lao, People's Democratic Republic, *P. falciparum* (95%) is also the major cause of malaria cases [19]. Some other studies show that there are three major environmental elements that influence the spread of malaria and the activity of mosquitoes: temperature (within 20-30°C), relative humidity (not below 60%), and rainfall (which increases relative humidity and modifies temperature), and Bangladesh is quite affected by these three factors [20-25]. From this study, we could not measure the rate and extent of the use of interventions like LLINs and RDT as there were inadequate data, but from an extensive literature review, it was found that RDT and LLINs were used for malaria diagnosis, control and prevention. Some other factors, such as improved education and awareness and improved socio-economic conditions, are included in the policies of the NMCP with regard to the control of malaria [6].

In order to achieve the goal of eliminating malaria, the National Malaria Control Program Bangladesh should take into consideration the following recommendations: (a) ensuring a robust surveillance and feedback process through alert monitoring of data collection; (b) creating strategies to impede local transmission by recognizing asymptomatic infections and successfully managing all infectious diseases before transmission can actually take place.

## Conclusions

The study concluded that although mortality is declining as the NMCP is functioning but the incidence is still high. The highest incidence rate among the five districts is in Rangamati; hence, there needs to be more monitoring and control for this. The findings show that Bangladesh will most likely achieve the goal of reducing malaria to low levels if the monitoring, evaluation, and surveillance systems operate properly along with treatment and other control interventions. The results show that the current control strategies are doing well, but the NMCP needs to focus on the proper use of interventions and combined work with other local and international organizations to ensure ongoing development in malaria prevention and eradication.

## References

[1] (2013). World Health Organization. World malaria report. https://www.cabdirect.org/cabdirect/abstract/20133318577.

[2] Snow RW, Craig M, Deichmann U, Marsh K (1999). Estimating mortality, morbidity and disability due to malaria among Africa's non-pregnant population. Bull World Health Organ.

[3] Sharma VP (2013). Re-emergence of malaria in India. Indian J Med Res.

[4] World Health Organization (1999). Hepatitis C—global prevalence (update). Wkly Epidemiol Rec.

[5] Wijeyaratne PM, Valecha N, Joshi AB, Singh D, Pandey S (2005). An inventory on malaria drug resistance in Bangladesh, Bhutan, India and Nepal. Environ Health Project Activity Report.

[6] (2006). National Malaria Control Program Bangladesh. https://old.dghs.gov.bd/index.php/en/component/content/article.

[7] Roll Back Malaria (2011). Refined/Updated GMAP Objectives, Targets, Milestones and Priorities Beyond 2011. https://scholar.google.com/scholar?hl=en&as_sdt=0%2C5&scioq=7.+World+Health+Organization.+Refined%2FUpdated+GMAP+Objectives%2C+Targets%2C+Milestones+and+Priorities+Beyond+2011.&q=7.%09World+Health+Organization.+Refined%2FUpdated+GMAP+Objectives%2C+Targets%2C+Milestones+and+Priorities+Beyond+2011.&btnG=.

[8] Bangladesh Statistics (2019). Bangladesh Bureau of Statistics (BSS). Retrieved J.

[9] Ngomane L, de Jager C (2012). Changes in malaria morbidity and mortality in Mpumalanga Province, South Africa (2001-2009): a retrospective study. Malar J.

[10] Mayosi BM, Lawn JE, Van Niekerk A (2009). Health in South Africa: changes and challenges since 2009. Lancet.

[11] Mohamedani AA, Mirgani EM, Ibrahim AM (1996). Gender aspects and women's participation in the control and management of malaria in central Sudan. Soc Sci Med.

[12] Govere JM, Durrheim DN, Coetzee M, Hunt RH (20011). Malaria in Mpumalanga Province, South Africa, with special reference to the period 1987-1999. South Afr J Sci.

[13] Kleinschmidt I, Sharp B (2001). Patterns in age-specific malaria incidence in a population exposed to low levels of malaria transmission intensity. Trop Med Int Health.

[14] Gerritsen AA, Kruger P, van der Loeff MF, Grobusch MP (2008). Malaria incidence in Limpopo Province, South Africa, 1998-2007. Malar J.

[15] Reuben R (19931). Women and malaria—special risks and appropriate control strategy. Soc Sci Med.

[16] Ghebreyesus TA, Witten KH, Getachew A, Yohannes AM, Tesfay W, Minass M, Bosman A (2000). The community-based malaria control programme in Tigray, northern Ethiopia. A review of programme set-up, outcomes and impact. Parassitologia.

[17] Hay SI, Okiro EA, Gething PW, Patil AP, Tatem AJ, Guerra CA, Snow RW (2010). Estimating the global clinical burden of Plasmodium falciparum malaria in 2007. PLoS Med.

[18] Dhiman S, Goswami D, Rabha B, Gopalakrishnan R, Baruah I, Singh L (2010). Malaria epidemiology along Indo-Bangladesh border in Tripura state, India. Southeast Asian J Trop Med Public Health.

[19] Jorgensen P, Nambanya S, Gopinath D (2010). High heterogeneity in Plasmodium falciparum risk illustrates the need for detailed mapping to guide resource allocation: a new malaria risk map of the Lao People's Democratic Republic. Malar J.

[20] Harlow J, Votava P, Running S (2001). Monitoring and Prediction of Malaria Outbreaks. Numerical Terradynamic Simulation Group.

[21] Thomson MC, Connor SJ (2001). The development of malaria early warning systems for Africa. Trends Parasitol.

[22] Yang HM (2000). Malaria transmission model for different levels of acquired immunity and temperature-dependent parameters (vector). Rev Saude Publica.

[23] Bi P, Tong S, Donald K, Parton KA, Ni J (2003). Climatic variables and transmission of malaria: a 12-year data analysis in Shuchen County, China. Public Health Rep.

[24] Mouchet JE, Manguin S, Sircoulon JA (1998). Evolution of malaria in Africa for the past 40 years: impact of climatic and human factors. J Am Mosquito Control Assoc.

[25] Pampana E (1969). A Textbook of Malaria Eradication. https://www.cabdirect.org/cabdirect/abstract/19702900209.

